# Novel Indolocarbazole Derivative 12-(*α*-*L*-arabinopyranosyl)indolo[2,3-*a*]pyrrolo[3,4-*c*]carbazole-5,7-dione Is a Preferred c-*Myc* Guanine Quadruplex Ligand

**DOI:** 10.4061/2011/184735

**Published:** 2011-05-19

**Authors:** Dmitry N. Kaluzhny, Anna K. Shchyolkina, Nikolay S. Ilyinsky, Olga F. Borisova, Alexander A. Shtil

**Affiliations:** ^1^Engelhardt Institute of Molecular Biology, Russian Academy of Sciences, 32 Vavilov Street, Moscow 119991, Russia; ^2^Moscow Institute of Physics and Technology, 9 Institutskii per., Dolgoprudny 141700, Russia; ^3^Blokhin Cancer Center, 24 Kashirskoye shosse, Moscow 115478, Russia

## Abstract

The indolocarbazole derivative 12-(*α*-*L*-arabinopyranosyl)indolo[2,3-*a*]pyrrolo[3,4-*c*]carbazole-5,7-dione (AIC) has demonstrated a high potency (at nanomolar to submicromolar concentrations) towards the NCI panel of human tumor cell lines and transplanted tumors. Intercalation into the DNA double helix has been identified as an important prerequisite for AIC cytotoxicity. In this study, we provide evidence for preferential binding to the G-quadruplex derived from the c-*Myc* oncogene promoter (Pu18 d(AG_3_TG_4_)_2_; G-c-*Myc*). The association constant for AIC:G-c-*Myc* complex was ~100 times and 10 times greater than the respective values for the complexes AIC:c-*Myc* duplex and AIC:telomeric d(TTAGGG)_4_ G-quadruplex. The concentrations at which AIC formed complexes with G-c-*Myc* were close to those that attenuated the steady-state level of the c-*Myc* mRNA in the human HCT116 colon carcinoma cell line. We suggest that preferential binding of AIC to G-c-*Myc* rather than to the c-*Myc* duplex might favor the quadruplex formation in the cells, thereby contributing to downregulation of the c-*Myc* expression by AIC.

## 1. Introduction

The c-*Myc* oncoprotein is an important transcription factor that plays a pivotal role in cell proliferation and survival [1]. Deregulation of c-*Myc* gene expression has been detected in many tumor types and is believed to be an important step in tumorigenesis [2]. A major control element of the human c-*Myc* gene is a purine/pyrimidine rich region (Pu27; the nuclease-hypersensitive element 1) located 115 bases upstream from the P1 promoter. This element controls up to 95% of the total c-*Myc* transcription [3, 4]. The Pu27 segment has been shown to adopt an intramolecular DNA tetraplex (G-quadruplex) conformation under physiological conditions [5]. The oligomer containing four consecutive 3′ runs of guanines d(AGGGTGGGGAGGGTGGGG) (Pu18) is the minimal prerequisite for the c-*Myc* G-quadruplex (G-c-*Myc*) formation [6, 7]. 

Involvement of the G-quadruplex structures in regulation of gene transcription [8, 9], RNA metabolism [10] and telomere function [11] opens an area for design of small molecular weight compounds that can preferentially interact with G-quadruplex DNA. The distinctive nature of quadruplex topologies suggests that quadruplex ligands may selectively silence a given gene [8] with little effect on other DNA regions containing noncanonical higher-order structures. A number of G-quadruplex ligands have been designed that demonstrated either similar affinity to various G-quadruplexes or preferential binding to a particular tetraplex. For example, the porphyrin derivative TMPyP4, the cyclic polyamine telomestatin, and 2,6-pyridin-dicarboxamide recognized both G-c-*Myc* and telomeric G-quadruplex (G-tel) [12, 13]. In contrast, fluoroquinolone derivative quarfloxin, the first therapeutic G-c-*Myc* ligand that entered clinical trials, did not show the cellular behavior characteristic of the telomere-targeting agent [11, 13]. The trisubstituted isoalloxazines specifically interacted with the *c-Kit *sequence and downregulated *c-Kit *oncogene expression [14].

**Figure 1:**
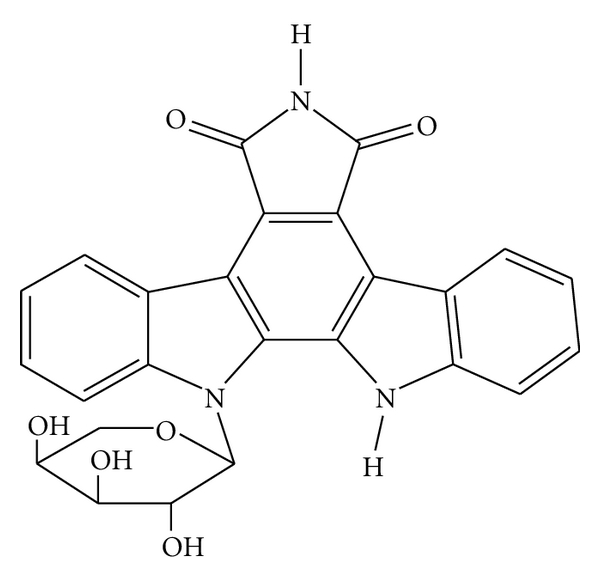
Structure of 12-(*α*-*L*-arabinopyranosyl)indolo[2,3-*a*]pyrrolo[3,4-*c*]carbazole-5,7-dione (AIC).

Indolocarbazoles and their congeners, a class of extensively investigated antitumor drugs, interact with multiple intracellular targets [15]. The mechanisms of antitumor effects of carbohydrate derivatives of indolocarbazoles include DNA damage and inhibition of the enzymes critical for cell viability, in particular, topoisomerase I, cyclin-dependent kinases, and the checkpoint kinase 1 [15–17]. The carbohydrate moiety of indolocarbazoles can enhance the binding of the conjugate to a target [16]. However, the binding constants to the duplex and G-quadruplex DNAs of indolocarbazoles containing one carbohydrate substituent are relatively low (*K*_*a*_ ≤ 2 · 10^5^ M^−1^) [18–20]. In contrast, we found the association constant to native double stranded DNA (dsDNA) of the novel indolocarbazole derivative, 12-(*α*-*L*-arabinopyranosyl)indolo[2,3-**α**]pyrrolo[3,4-*c*]carbazole-5,7-dione (AIC, Figure 1) to be substantially higher (1.6·10^6^ M^−1^ ≤ *K*_*a*_ ≤ 3.3·10^6^ M^−1^) [21, 22]. AIC potently inhibited growth of 60 cultured human tumor cell lines (National Cancer Institute drug screening panel). This compound demonstrated promising in vivo therapeutic efficacy against transplanted tumors. The concentrations of AIC required for the formation of intercalative complexes with dsDNA and the concentrations that induced tumor cell death were similar, suggesting that intercalation into DNA is an important factor of cytotoxicity of this indolocarbazole derivative [22].

In this study we analyzed the interactions of AIC with G-quadruplex DNA structures. We explored the affinity and selectivity of AIC interaction with the intramolecular G-c-*Myc* formed by Pu18 [6]. The association constants of AIC:G-c-*Myc* complexes were exceptionally high, ~100 times greater than the respective value for the complexes of AIC with 18-bp c-*Myc* duplex DNA. The concentrations at which AIC formed complexes with G-c-*Myc* were close to the concentrations that attenuated the steady-state level of the c-*Myc* mRNA in the human HCT116 colon carcinoma cell line.

## 2. Material and Methods

### 2.1. Reagents and Sample Preparation

All reagents were from Sigma-Aldrich, St. Louis, Mo unless otherwise specified. The oligonucleotides d(AG_3_TG_4_)_2_ (G-c-*Myc*), d(C_4_AC_3_T)_2_ (comp-c-*Myc*) and d(TTAGGG)_4_ (G-tel) were synthesized by Syntol (Moscow). The DNA preparations were dissolved in Hanks' buffer (0.137 M NaCl, 5.4 mM KCl, 0.25 mM Na_2_HPO_4_, 0.44 mM KH_2_PO_4_, 1.3 mM CaCl_2_, 1 mM MgSO_4_, 4.2 mM NaHCO_3_, pH 7.2) immediately before the experiments. The molar extinction coefficient of AIC was *ε*_320_ = 22 000 M^−1^. All experiments were performed in Hanks' buffer at 20°C.

### 2.2. Instruments

Absorption spectra were acquired with a Jasco V-550 spectrophotometer (Japan). Fluorescence was registered with a Cary Eclipse spectrofluorimeter (Varian Inc.). CD spectra were recorded using a Jasco 715 spectropolarimeter (Japan). Molar dichroism (Δ*ε*) was calculated per mole of nucleotides. All instruments were equipped with thermostated cell holders.

### 2.3. Binding of AIC to DNA

The binding of AIC to various DNAs was monitored by UV light absorption and spectrofluorimetry [23]. The AIC:DNA complex formation was represented by Scatchard plots, that is, by the ratio *r*/*C*_1_ as the function of *r*, where *C*_1_ is concentration of free AIC, and *r* is the number of bound AIC molecules per G-quadruplex or DNA duplex. The experimental data were fitted by (1) for several independent types of bound molecules:

(1) $$r=\overset{N}{\underset{i=1}{\sum }}\frac{n_{i}K_{i}C_{1}}{1+K_{i}C_{1}},$$

where *N* is the number of independent types of binding, *n*_*i*_ is the maximal number of molecules that participate in one type of binding, *K*_*i*_ is the association constant of *i* type of complex, and *C*_1_ is the concentration of unbound AIC.

Studies of energy transfer and circular dichroism (CD) spectra were performed as described by Kaluzhny et al. [22].

### 2.4. Measurement of c-Myc mRNA by Quantitative Real-Time PCR

The HCT116 human colon carcinoma cell line (American Type Culture Collection, Manassas, Va) was cultured in RPMI-1640 supplemented with 5% fetal calf serum (HyClone, Logan, Ut), 2 mM *L*-glutamine, 100 U/mL penicillin, and 100 *μ*g/mL streptomycin at 37°C, 5% CO_2_ in a humidified atmosphere. Cells (2 × 10^5^ in 6 mL of culture medium) were plated onto 60 mm Petri dishes overnight. Then AIC was added, and the cells were incubated for 24–48 h. An Agilent Total RNA mini-isolation kit (Agilent, Tex) was used for RNA isolation. Reverse transcription was performed as previously described [24]. For quantitative real-time PCR, cDNA samples were amplified with primers corresponding to c-*Myc* (forward 5′-AAAGACAGCGGCAGCCCGAA-3′; reverse 5′-CCAAGTCCTGCGCCTCGCAAG-3′) and glyceraldehyde-3-phosphate dehydrogenase (GAPDH) (forward, 5′-GAAGGTCGGAGTCAACGGATT-3′; reverse 5′-GCCAAAAGGGTCATCATCTCT-3′). The reactions were performed using Stratagene Brilliant II SYBR Green QPCR Master Mix on a Stratagene MX 3005P thermocycler. The MxPro (QPCR Software) was used for data analysis. The amounts of c-*Myc* mRNA in AIC-treated cells were expressed as the percentage of the signal intensity to that in control (untreated) samples (regarded as 100%).

## 3. Results

### 3.1. UV Absorption of AIC:DNA Complexes

**Figure 2:**
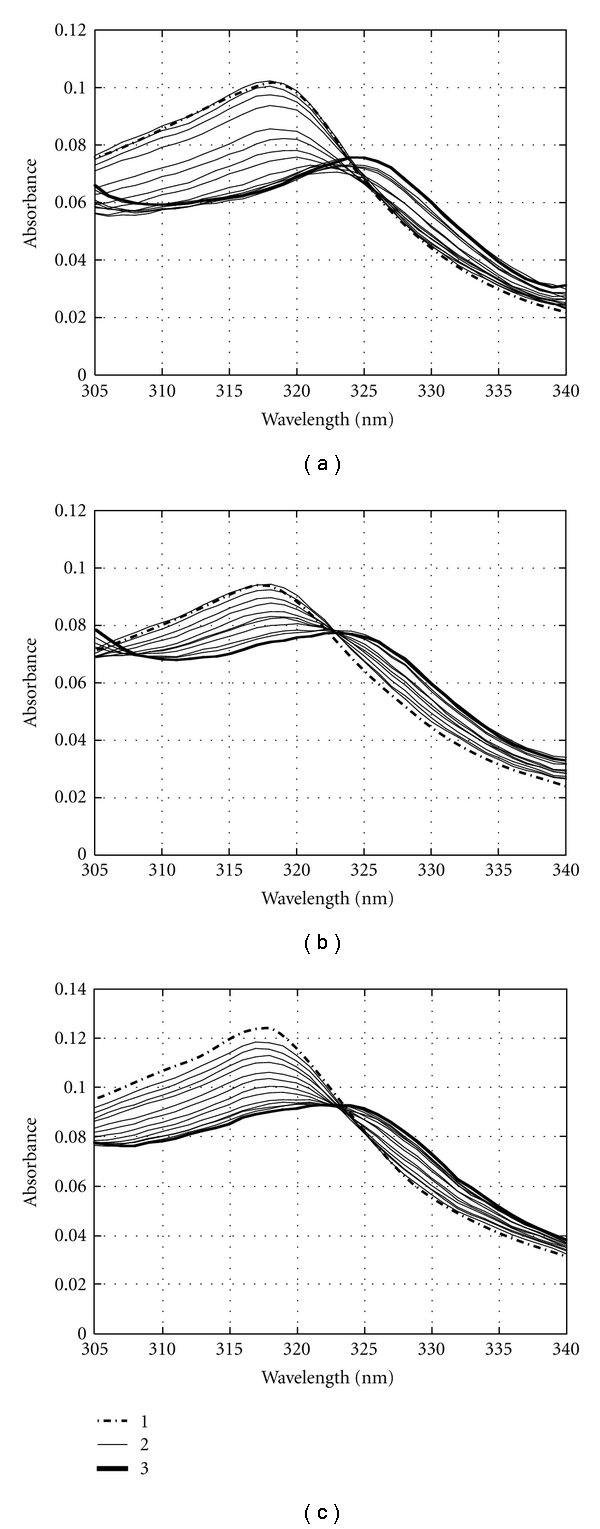
UV absorption spectra of AIC:DNA complexes: (a) AIC:G-c-*Myc*, (b) AIC:G-tel, and (c) AIC:c-*Myc* duplex. The concentrations of oligomers Pu18 and d(TTAGGG)4 were 0–2.5 *μ*M (strands) and of c-*Myc* duplex were 0–3 *μ*M (strands). Curves 1–3 indicate the spectra of free AIC, partially bound AIC and fully bound AIC, respectively: Hanks' buffer, *t* = 20°C.

**Figure 3:**
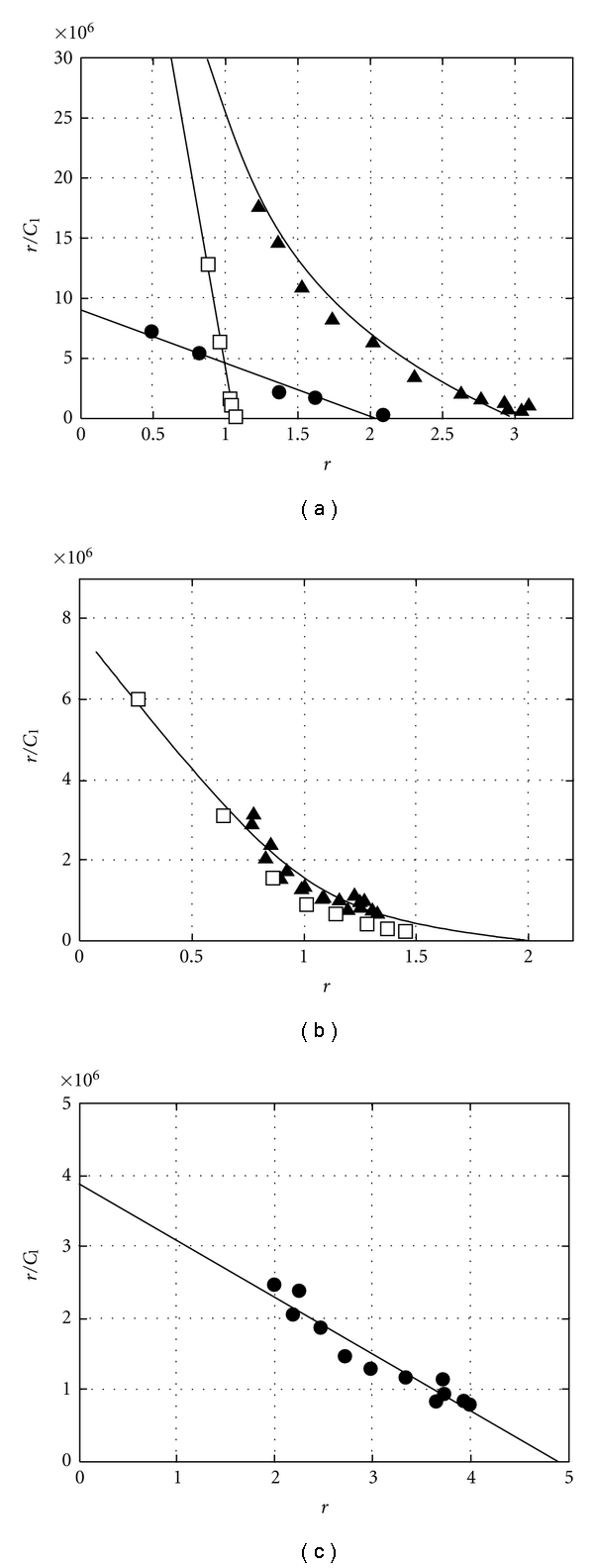
Scatchard plots of AIC:DNA binding. (a) The curve of AIC binding to G-c-*Myc* (Pu18) registered by UV absorption (triangles) and spectrofluorimetry (squares). The circles represent absorption of nonfluorescent complexes. (b) The curve of AIC binding to G-tel registered by UV absorption (triangles) and spectrofluorimetry (squares). (c) The curve of AIC binding to Pu18 c-*Myc* duplex registered by UV absorption. The parameters *r* and *r*_*f*_ were calculated per DNA strand: Hanks' buffer, *t* = 20°C.

The UV absorption spectra of free AIC showed two prominent bands at 285 nm and 318 nm and a weaker band at 425 nm [22]. The absorption near *λ* = 318 nm decreased upon binding of AIC to G-c-*Myc*, G-tel, and c-*Myc* duplex d(AGGGTGGGG)_2_:d(CCCCACCCT)_2_ in Hanks' buffer at 20°C (Figure 2). The absorption maximum shifted from 318 nm to ~325 nm. The spectral changes were similar to those observed for AIC intercalation into dsDNA [22]. Using the absorption at *λ* = 318 nm we plotted the AIC-binding curves to G-c-*Myc*, G-tel, and c-*Myc* duplex in Scatchard coordinates, *r*/*C*_1_ on *r* (Figure 3, triangles). Based on these curves we calculated maximal numbers of AIC molecules bound to the oligonucleotides: three molecules to G-c-*Myc*, two to G-tel, and five to the c-*Myc* duplex.

### 3.2. Fluorescence of AIC:G-Quadruplex Complexes

**Figure 4:**
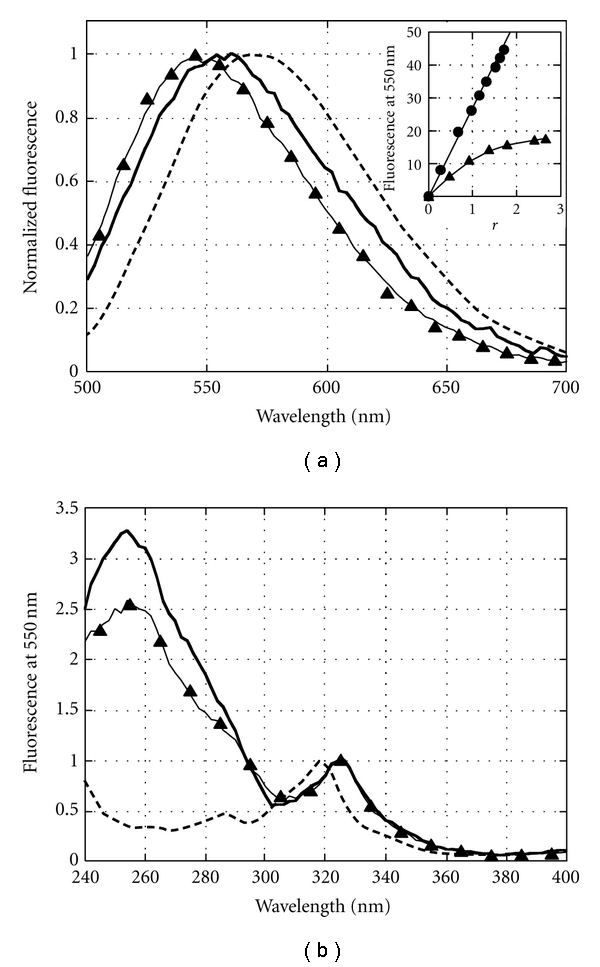
Fluorescence of AIC:G-quadruplex complexes. (a) The fluorescence spectra of free AIC in 95% ethanol solution (dashed line), AIC bound to Pu18 G-c-*Myc *(solid line) and to G-tel (triangles). The intensity of fluorescence was registered after excitation at 320 nm and normalized to 1. The parameter *r* was ~1 for all complexes. Inset: fluorescence of AIC:G-quadruplex complexes depending on *r*. Complexes AIC:G-c-*Myc *(triangles), AIC:G-tel (circles). (b) Excitation spectra of complexes AIC:G-c-*Myc *(solid line), AIC:G-tel (triangles), and free AIC in 95% ethanol solution (dashed line). Fluorescence intensity *I*_550_ registered at 320 nm was normalized to 1. The numbers of bound AIC molecules per G-c-*Myc* and G-tel were *r* = 1.

Fluorescence emission at 500 < *λ* < 650 nm centered at ~550 nm was detectable upon binding of AIC to G-c-*Myc* (Figure 4(a), solid curve) and to G-tel (Figure 4(a), triangles). Similar fluorescence emission was typical for AIC intercalation into dsDNA [22]. In aqueous solution the fluorescence of the drug was quenched [20]. Therefore, the fluorescence spectrum of free AIC in ethanol was taken for comparison with the respective spectra of complexes AIC:G-c-*Myc* and AIC:G-tel (Figure 4(a), dashed curve). 

The dependencies of fluorescence intensities (*I_550_*) of AIC:G-c-*Myc* and AIC:G-tel on *r* value (calculated using the binding curves in Figures 3(a) and 3(b); triangles) were plotted in Figure 4(a), *inset *(triangles and circles, resp.). The fluorescence intensity of AIC:G-c-*Myc* complexes increased linearly with the concentration of bound AIC (0 < *r* ≤ 1). The fluorescence intensity increased much more slowly at *r* > 1 (note ~10-fold smaller slope of the curve). Thus, the fluorescence of the second and the third bound AIC molecules (1 < *r* ≤ 3) quenched significantly. In contrast, the fluorescence intensity of AIC:G-tel grew linearly at 0 < *r* ≤ 2. Apparently, two AIC molecules bound to G-tel fluoresced with the same quantum yield. 

These results strongly suggest that only the first AIC molecule tightly bound to G-c-*Myc* is fluorescent being placed in a hydrophobic environment. Such a highly fluorescent AIC molecule may stack between the terminal G-quartet and 5′-adenine [7]. Two other AIC molecules whose fluorescence is markedly quenched are supposed to bind to the two propeller loops in a less hydrophobic environment. One may hypothesize that the fluorescence of AIC bound to the loops might be quenched by water molecules. Two hydrophobic binding sites are present in the antiparallel G-tel. The AIC molecules may be stacked between each of the terminal G-quartets and the bases of lateral and diagonal loops across the quartets [25]. The structures of the two sites are not identical; therefore, the binding curve is nonlinear (Figure 3(b), triangles).

### 3.3. Parameters of AIC Binding to Various DNA Structures

We generated the Scatchard plot (*r*_*f*_/*C*_1_ on *r*_*f*_) for the binding of fluorescent AIC molecules to G-c-*Myc* (Figure 3(a), squares). The *C*_1_ values were derived from the binding curve drawn on the basis of UV absorption data (Figure 3(a), triangles). The binding curve revealed a single “strong” binding site, *K*_*f*_ = (7.0 ± 1) · 10^7^ M^−1^. We subtracted the contribution of the fluorescent molecules (Figure 3(a), squares) from the binding curve constructed using UV absorption (Figure 3(a), triangles). The resulting Scatchard plot revealed the two quenched ligand molecules with the association constant *K*_2_ = (4 ± 1) · 10^6^ M^−1^ (Figure 3(a), circles). The curve in Figure 2(a), triangles, reflects the formation of two types of complexes, that is, the fluorescent *K*_1_ = (7 ± 1) · 10^7^ M^−1^, *n*_1_ = 1 and the nonfluorescent (quenched) *K*_2_ = (4 ± 1) · 10^6^ M^−1^, *n*_2_ = 2, respectively (Table 1). This curve was analyzed using a model for two independent types of drug-binding sites (1). The theoretical plot corresponding to the *K*_1_, *K*_2_, *n*_1_, *n*_2_ values (Table 1) is shown in Figure 2(a), solid curve. 

The curve of AIC binding to the G-tel generated using the fluorimetric method (Figure 3(b), squares) coincided with the binding curve obtained by UV absorption (Figure 3(b), triangles). The binding curve may be approximated with two independent binding sites. The calculated association constants are given in Table 1. The association constants for the strong and the weak AIC:G-tel complexes were ~10 times lower than those for AIC:G-c-*Myc* complexes (Table 1).

The Scatchard plot for AIC binding to the 18 bp c-*Myc* duplex (generated by UV absorption) was linear at 2 ≤ *r* ≤ 4 (Figure 3(c). The binding curve was approximated with the equation for a single type complex. The 18 bp c-*Myc* duplex formed complexes with five inner AIC molecules, *K* = (8 ± 1) · 10^5^ M^−1^ (Table 1). The association constant is close to that for strong AIC binding to dsDNAs of various nucleotide content 1.6 · 10^6^ < *K*_1_ ≤ 3.3 · 10^6^ M^−1^) [22]. Importantly, the affinity of AIC to c-*Myc* duplex appeared to be ~100 times weaker than to G-c-*Myc* (Table 1). Hence, the G-quadruplex conformation(s) may be energetically favorable for binding of AIC to DNA within the c-*Myc *regulatory region. Altogether, G-c-*Myc* emerges as a preferred target for AIC.

### 3.4. Energy Transfer from G-Quadruplexes to AIC

To investigate whether the stacking contacts and intercalation might be the modes of AIC:G-quadruplex complex formation, we studied the energy transfer from the bases of G-c-*Myc* or G-tel to bound AIC molecules. Because the unbound AIC molecules fluoresce only negligibly in aqueous solution, the excitation spectra of free AIC in ethanol were taken for comparison with the respective spectra of complexes AIC:G-c-*Myc* and AIC:G-tel. The excitation spectra of AIC:G-c-*Myc*, AIC:G-tel, and the free drug in ethanol are shown in Figure 4(b). The fluorescence intensity *I*_550_ of AIC was registered upon excitation in the region 240 < *λ* < 340 nm. All spectra were normalized with respect to the fluorescence intensity *I*_550_ corresponding to excitation at *λ* = 320 nm. The occupancy value *r* for AIC:G-c-*Myc* and AIC:G-tel was ~1, that is, the AIC concentration was low enough for predominant formation of strong AIC:G-quadruplex complexes. Virtually no excitation signal of free AIC was detectable at ~260 nm (Figure 4(b), dashed line). The excitation spectra of AIC:G-quadruplexes demonstrated a markedly increased fluorescence at 250 < *λ* < 280 nm, the region of nucleotide absorption (Figure 4(b), bold curve and triangles). The signal in the region 250 < *λ* < 280 nm in the excitation spectra of AIC:G-quadruplexes indicated the energy transfer from the bases of G-quadruplexes to AIC. This effect is known to occur if a planar molecule is involved in stacking interactions with the adjacent bases. In contrast, no energy transfer is detectable when fluorescent molecules bind to the DNA grooves [20, 26]. Thus, the energy transfer data supported the stacking interaction of AIC with nucleotides, possibly involving an end stacking to terminal G-quartets in G-c-*Myc* and G-tel.

### 3.5. Circular Dichroism Spectra of AIC:G-c-Myc Complexes

**Figure 5:**
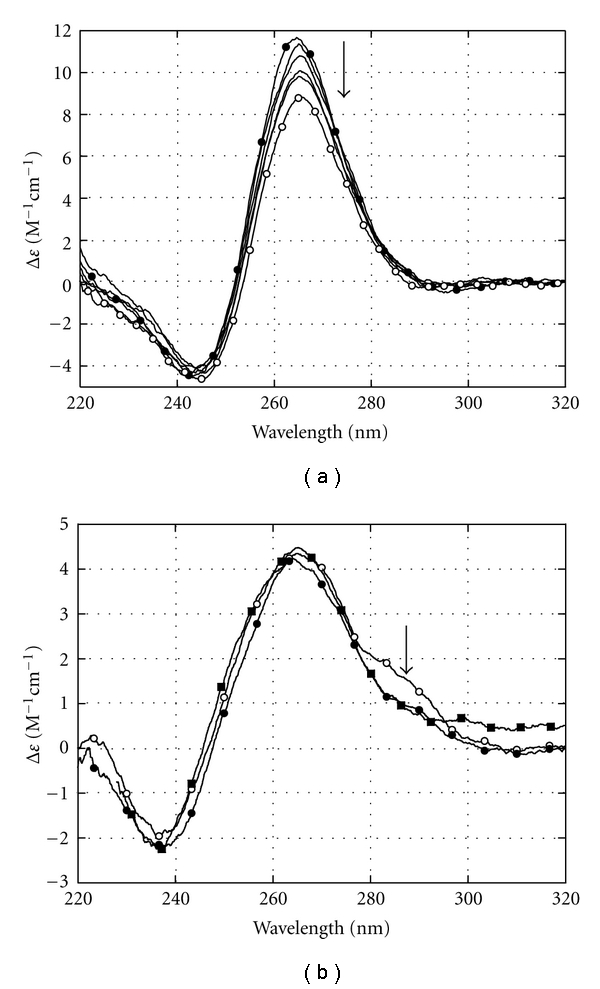
CD spectra of G-c-*Myc* and c-*Myc *duplex at different AIC concentrations: (a) CD spectra of free G-c-*Myc* (filled circles) and in complex with 3 AIC molecules (open circles). The concentration of G-c-*Myc* was 1 *μ*M; the concentrations of AIC corresponded to *r* = 0; 0.5; 1.14; 2.0; 2.5; ~3.0 Hanks' buffer, *t* = 20°C. (b) CD spectra of Pu18 c-*Myc *duplex (0.5 *μ*M): free (open circles), in complexes with 3 AIC molecules (filled circles) and in the presence of an excess of AIC (12 molecules; filled squares).

We detected no induced CD signal in the wavelength region of AIC absorption upon binding of the drug to the oligonucleotides studied. To monitor the changes of the Pu18 G-c-*Myc* conformation upon AIC binding, we registered CD spectra of the complexes in the wavelength region of nucleotide absorption (220 < *λ* < 320 nm) (Figure 5). The parameters of CD spectra proved that free G-c-*Myc* adopted the structure of a parallel Pu18 G-quadruplex [6]. The CD spectra of G-c-*Myc* changed upon binding to AIC. The magnitude of the positive CD band at *λ* = 265 nm decreased after the addition of the drug. At maximal occupancy, *r* = 3, the magnitude of the band decreased by ~25%. These results may be interpreted as a reduction of stacking contacts between G-quartets. Nevertheless, the parallel conformation of the quadruplex remained intact upon drug binding. 

We performed CD experiments to reveal whether AIC binding induces c-*Myc* duplex dissociation followed by G-quadruplex formation. The CD spectrum of the sample (Figure 5(b), open circles) is typical for a double-stranded oligonucleotide rich in G-runs. Only small changes around 280–290 nm occurred upon binding of 3 AIC molecules per duplex (Figure 5(b), filled circles). The excess of AIC (12 molecules per duplex) did not affect the CD spectrum of the oligonucleotide, however, a positive CD signal above 320 nm was detectable (Figure 5(b), filled squares). This induced CD band may reflect the interactions of AIC molecules with each other and with DNA bases. No changes in the duplex conformation were detected upon AIC binding. Thus, AIC binding to c-*Myc* duplex induced no structural rearrangements such as duplex dissociation and G-c-*Myc* formation. Still, these results do not presume that AIC cannot affect the duplex-quadruplex equilibrium in the cell.

### 3.6. Downregulation of c-Myc Expression by AIC

**Figure 6:**
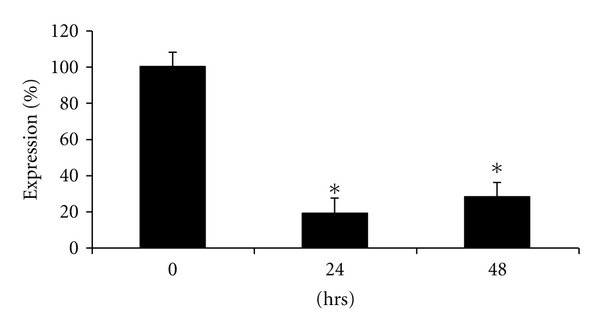
Downregulation of steady-state level of c-*Myc* mRNA by AIC. See text for details. Data are mean + S.D. of 3 independent experiments. *P < .01 compared with control values.

To reveal whether AIC, a strong G-c-*Myc* ligand (see above), can modulate the steady-state level of c-*Myc* mRNA in living cells, we analyzed c-*Myc* transcripts in HCT116 human colon carcinoma cell line by real-time PCR after reverse transcription. As shown in Figure 6, the amounts of c-*Myc* mRNA decreased in cells treated with 1 *μ*M of AIC for 24–48 h. By 24 h the amount of c-*Myc* mRNA was 4–6 times smaller than that in untreated cells. The decreased level of c-*Myc* transcripts remained sustained for up to 48 h of exposure (Figure 6). In contrast, no effect on in vitro telomerase activity was detectable even at concentrations of AIC up to 10 *μ*M (data not shown). These results are in line with preferential affinity of AIC to G-c-*Myc* rather than to G-tel.

## 4. Discussion

Previously we have shown a high potency of AIC in a panel of human tumor cell lines and in vivo tumor models [22]. The duplex DNA, in particular, DNA breaks have been identified as intracellular AIC targets important for cytotoxicity of this indolocarbazole derivative. In the present study we identified G-c-*Myc* as a new selective target of AIC. The high affinity of AIC to G-c-*Myc* (*K*_1_ = 7 ± 1 · 10^7^ M^−1^) was ~100 times greater than that to the c-*Myc* derived duplex DNA. AIC demonstrated a ~10-fold preference for the parallel G-c-*Myc* conformation over the antiparallel G-tel (Table 1). Accordingly, treatment of HCT116 cells with 1 *μ*M of AIC attenuated the amount of c-*Myc* mRNA whereas the in vitro telomerase activity remained unaltered even by 10 *μ*M of the drug.

Design of therapeutic G-quadruplex binding drugs may be based on differential structure of individual tetraplex DNAs and/or on simultaneous targeting of quadruplexes by a particular ligand. A structural preference for the parallel c-*Myc* or c-*Kit *quadruplexes over the antiparallel G-tel has been reported for several ligands [14, 27, 28]. Taking advantage of different degrees of structural plasticity of parallel and antiparallel quadruplexes in binding ligands, Garner et al. have identified an extended heteroaromatic 1,4-triazole (TRZ) as a compound with some selectivity for parallel folds whereas the polycyclic fluorinated acridinium cation RHPS4 showed selectivity for antiparallel conformations [29]. However, RHPS4 and TRZ were similarly potent telomerase inhibitors [29]. Interestingly, the porphyrin derivative 5,10,15,20-tetrakis(1-methyl-4-pyridyl)-21H, 23H-porphine (TMPyP4) demonstrated the properties similar to AIC. The binding parameters of TMPyP4 to G-c-*Myc* at ionic strength K^+^ = 0.1 M (*K*_1_ = 7 ± 1 · 10^7^ M^−1^, *n*_1_ = 1 and *K*_2_ = 1 · 10^6^ M^−1^, *n*_2_ = 2) [28] were close to AIC binding to G-c-*Myc* Pu18 in Hanks' buffer (Table 1). Furthermore, TMPyP4 preferentially bound to G-c-*Myc *rather than to c-*Myc* duplex DNA [28]. Tight TMPyP4 binding sites were suggested to be at the terminal G-quartets while the weaker binding sites were located at the nucleotide loops [6, 27]. Our data suggest that binding of AIC to G-c-*Myc *may occur via similar sites. The signal in the region 250 < *λ* < 280 nm of the excitation spectra of AIC:G-c-*Myc *complexes indicated energy transfer from the bases of G-quadruplexes to AIC. Thus, the energy transfer data supported the mode of AIC:nucleotide interactions, most likely involving an end stacking to terminal G-quartets in G-c-*Myc* and G-tel. 

Although much of the structural work on G-quadruplexes has been done in vitro, evidence is growing to prove the roles of these structures in vivo [30, 31]. The formation of G-quadruplexes is facilitated by negative superhelical stress produced during transcription and can be stabilized by monovalent cations such as K^+^ and Na^+^ that intercalate between the G-tetrads and coordinate the bonds with guanine carbonyl groups. Transcription-associated supercoiling has been demonstrated to evoke dynamic effects on DNA conformation in the c-*Myc *regulatory region: the duplex DNA can be converted into nonduplex DNA, even at considerable distances from the transcriptional start site [32]. These nonduplex DNA structures that control both transcriptional switch on/off and the rate of transcription firing, are amenable to targeting by small molecular weight ligands. The fact that AIC is a strong ligand not only for c-*Myc*-derived G-quadruplex but also for c-*Myc *duplex (notwithstanding the preference of the former conformational structure) implies that this ligand might be relevant for treatment of c-*Myc* positive tumors due to the ability to interact with various regions within this oncogene. One can hypothesize that the formation of drug-DNA complexes at multiple sites is linked to a decrease of c-*Myc* mRNA in AIC-treated cells (see below).

The conformational transition from a duplex to a G-quadruplex within the c-*Myc* promoter has been proposed as a mechanism of c-*Myc* inhibition by TMPyP4 [27, 28]. Accordingly, the high selectivity of AIC for G-c*-Myc* may lead to prevalence of the quadruplex over the duplex DNA conformation in the cell. We believe that the formation of high affinity complexes AIC:G-c-*Myc* is likely to be a prerequisite for downregulation of c-*Myc* expression. Still, drug-quadruplex interaction may not be the sole mechanism of c-*Myc* silencing by AIC. Other events including the interference with the formation of the transcriptional complexes and/or the traverse of the transcriptional machinery along the DNA template, as well as deregulation of mRNA stability, cannot be ruled out at this stage of the study. Moreover, dual targeting by a quadruplex ligand might be therapeutically beneficial. Indeed, the effect of the G-c-*Myc* ligand quarfloxin involves disruption of the interaction between ribosomal DNA G-quadruplexes and nucleolin [11, 13]. Gunaratnam et al. have identified a naphthalene diimide derivative as a compound that recognized both G-tel DNA and the quadruplex sites in the c-*Kit* promoter. This compound inhibited telomerase activity and suppressed c-*Kit* mRNA and protein expression, the effects associated with the induction of growth arrest in a patient-derived gastrointestinal stromal tumor cell line [33]. Whatever the mechanism(s), the identification of AIC as a preferred binder to c-*Myc* promoter-derived G-quadruplex may expand the therapeutic perspective of indolocarbazole derivatives. Together with the confirmed ability to target some protein kinases and dsDNA, these compounds emerge as high affinity ligands for noncanonical DNA structures and potential modulators of gene expression, tentatively via DNA conformation-dependent regulatory mechanisms.

## Figures and Tables

**Table 1:** Binding parameters of AIC:DNA complexes.

DNA	^ [a]^ *K*_1_ (×10^−6^), M^−1^	^ [b]^ *n*_1_	*K*_2_ (×10^−6^), M^−1^	*n*_2_
G-c-*Myc *	70 ± 10	1	4 ± 1	2
G-tel	6.5 ± 1.0	1	0.33 ± 0.05	1
c-*Myc *duplex	0.8 ± 0.1	5	—	—

[a]: *K*_1_, *K*_2_ are the association constants of AIC:DNA complexes;

[b]: *n*_1_, *n*_2_ are the maximal numbers of AIC molecules bound to a particular DNA.

## References

[1] Qin Y, Hurley LH (2008). Structures, folding patterns, and functions of intramolecular DNA G-quadruplexes found in eukaryotic promoter regions. *Biochimie*.

[2] Nesbit CE, Tersak JM, Prochownik EV (1999). MYC oncogenes and human neoplastic disease. *Oncogene*.

[3] Berberich SJ, Postel EH (1995). PuF/NM23-H2/NDPK-B transactivates a human c-myc promoter-CAT gene via a functional nuclease hypersensitive element. *Oncogene*.

[4] Davis TL, Firulli AB, Kinniburgh AJ (1989). Ribonucleoprotein and protein factors bind to an H-DNA-forming c-myc DNA element: possible regulators of the c-myc gene. *Proceedings of the National Academy of Sciences of the United States of America*.

[5] Simonsson T, Pecinka P, Kubista M (1998). DNA tetraplex formation in the control region of c-*myc*. *Nucleic Acids Research*.

[6] Seenisamy J, Rezler EM, Powell TJ (2004). The dynamic character of the G-quadruplex element in the c-MYC promoter and modification by TMPyP4. *Journal of the American Chemical Society*.

[7] Ambrus A, Chen D, Dai J, Jones RA, Yang D (2005). Solution structure of the biologically relevant G-quadruplex element in the human c-MYC promoter. Implications for G-quadruplex stabilization. *Biochemistry*.

[8] Balasubramanian S, Neidle S (2009). G-quadruplex nucleic acids as therapeutic targets. *Current Opinion in Chemical Biology*.

[9] Huppert JL (2008). Hunting G-quadruplexes. *Biochimie*.

[10] Melko M, Bardoni B (2010). The role of G-quadruplex in RNA metabolism: involvement of FMRP and FMR2P. *Biochimie*.

[11] Neidle S (2010). Human telomeric G-quadruplex: the current status of telomeric G-quadruplexes as therapeutic targets in human cancer. *FEBS Journal*.

[12] Lemarteleur T, Gomez D, Paterski R, Mandine E, Mailliet P, Riou JF (2004). Stabilization of the c-myc gene promoter quadruplex by specific ligands’ inhibitors of telomerase. *Biochemical and Biophysical Research Communications*.

[13] Drygin D, Siddiqui-Jain A, O’Brien S (2009). Anticancer activity of CX-3543: a direct inhibitor of rRNA biogenesis. *Cancer Research*.

[14] Bejugam M, Sewitz S, Shirude PS, Rodriguez R, Shahid R, Balasubramanian S (2007). Trisubstituted isoalloxazines as a new class of G-quadruplex binding ligands: small molecule regulation of c-kit oncogene expression. *Journal of the American Chemical Society*.

[15] Salas JA, Méndez C (2009). Indolocarbazole antitumour compounds by combinatorial biosynthesis. *Current Opinion in Chemical Biology*.

[16] Prudhomme M (2004). Biological targets of antitumor indolocarbazoles bearing a sugar moiety. *Current Medicinal Chemistry—Anti-Cancer Agents*.

[17] Nakano H, Omura S (2009). Chemical biology of natural indolocarbazole products: 30 years since the discovery of staurosporine. *Journal of Antibiotics*.

[18] Ren J, Bailly C, Chaires JB (2000). NB-506, an indolocarbazole topoisomerase I inhibitor, binds preferentially to triplex DNA. *FEBS Letters*.

[19] Bailly C, Qu X, Anizon F, Prudhomme M, Riou JF, Chaires JB (1999). Enhanced binding to DNA and topoisomerase I inhibition by an analog of the antitumor antibiotic rebeccamycin containing an amino sugar residue. *Molecular Pharmacology*.

[20] Bailly C, Qu X, Graves DE, Prudhomme M, Chaires JB (1999). Calories from carbohydrates: energetic contribution of the carbohydrate moiety of rebeccamycin to DNA binding and the effect of its orientation on topoisomerase I inhibition. *Chemistry and Biology*.

[21] Kalyuzhnyi DN, Tatarsk VV, Bondarev FS (2006). Interaction with DNA as a cytotoxicity factor of a novel glycoside derivative of indolocarbazole. *Doklady Biochemistry and Biophysics*.

[22] Kaluzhny DN, Tatarskiy VV, Dezhenkova LG (2009). Novel antitumor L-arabinose derivative of indolocarbazole with high affinity to DNA. *ChemMedChem*.

[23] Lepecq JB, Paoletti C (1967). A fluorescent complex between ethidium bromide and nucleic acids. physical-chemical characterization. *Journal of Molecular Biology*.

[24] Shtil AA, Mandlekar S, Yu R (1999). Differential regulation of mitogen-activated protein kinases by microtubule-binding agents in human breast cancer cells. *Oncogene*.

[25] Patel DJ, Phan AT, Kuryavyi V (2007). Human telomere, oncogenic promoter and 5′-UTR G-quadruplexes: diverse higher order DNA and RNA targets for cancer therapeutics. *Nucleic Acids Research*.

[26] Suh D, Chaires JB (1995). Criteria for the mode of binding of DNA binding agents. *Bioorganic and Medicinal Chemistry*.

[27] Seenisamy J, Bashyam S, Gokhale V (2005). Design and synthesis of an expanded porphyrin that has selectivity for the c-MYC G-quadruplex structure. *Journal of the American Chemical Society*.

[28] Arora A, Maiti S (2008). Effect of loop orientation on quadruplex-TMPyP4 interaction. *Journal of Physical Chemistry B*.

[29] Garner TP, Williams HEL, Gluszyk KI (2009). Selectivity of small molecule ligands for parallel and anti-parallel DNA G-quadruplex structures. *Organic and Biomolecular Chemistry*.

[30] Gonzalez V, Hurley LH (2010). The c-MYC NHE III: function and regulation. *Annual Review of Pharmacology and Toxicology*.

[31] Fry M (2007). Tetraplex DNA and its interacting proteins. *Frontiers in Bioscience*.

[32] Brooks TA, Hurley LH (2009). The role of supercoiling in transcriptional control of MYC and its importance in molecular therapeutics. *Nature Reviews Cancer*.

[33] Gunaratnam M, Swank S, Haider SM (2009). Targeting human gastrointestinal stromal tumor cells with a quadruplex-binding small molecule. *Journal of Medicinal Chemistry*.

